# Electroionic Antagonistic Muscles Based on Nitrogen‐Doped Carbons Derived from Poly(Triazine‐Triptycene)

**DOI:** 10.1002/advs.201700410

**Published:** 2017-10-11

**Authors:** Sandipan Roy, Jaehwan Kim, Moumita Kotal, Kwang Jin Kim, Il‐Kwon Oh

**Affiliations:** ^1^ Creative Research Initiative Center for Functionally Antagonistic Nano‐Engineering Department of Mechanical Engineering Korea Advanced Institute of Science and Technology (KAIST) 291 Daehak‐ro Daejeon Yuseong‐gu 34141 Republic of Korea; ^2^ Active Materials and Smart Living Laboratory Department of Mechanical Engineering University of Nevada Las Vegas (UNLV) Las Vegas NV 89154 USA

**Keywords:** actuator, artificial muscle, covalent organic framework, hierarchically porous nitrogen doped carbon, specific capacitance

## Abstract

Electroactive soft actuators and bioinspired artificial muscles have received burgeoning interest as essential components in future electronic devices such as soft haptic‐feedback systems, human‐friendly wearable electronics, and active biomedical devices. However, important challenging issues including fast response time, ultralow input power, robust operation in harsh environments, high‐resolution controllability, and cost‐effectiveness remain to be resolved for more practical applications. Here, an electroionic antagonistic artificial muscle is reported based on hierarchically porous nitrogen‐doped carbon (HPNC) electrodes derived from a microporous poly(triazine‐triptycene) organic framework (PtztpOF). The HPNC, which exhibits hierarchically micro‐ and mesoporous structures, high specific capacitance of 330 F g^−1^ in aqueous solution, large specific surface area of 830.46 m^2^ g^−1^, and graphitic nitrogen doping, offers high electrical conductivity of 0.073 MS m^−1^ and outstanding volumetric capacitance of 10.4 MF m^−3^. Furthermore, it is demonstrated that a novel electroionic antagonistic muscle based on HPNC electrodes successfully displays extremely reliable and large bending deformations and long‐term durability under ultralow input voltages. Therefore, microporous polymer or covalent organic frameworks can be applied to provide significant improvements in electroactive artificial muscles, which can play key roles as technological advances toward bioinspired actuating devices required for next‐generation soft and wearable electronics.

## 1 Introduction

Recently, electromechanical and electro‐chemomechanical artificial muscles that convert electrical signals to mechanical deformation have attracted intensive attention for potential applications in soft robotics, bioinspired operations, man‐machine haptic interfaces, curvature‐controllable flexible display, loudspeakers, and underwater sonar.^1^, ^2^, ^3^, ^4^, ^5^, ^6^ In particular, among the electroactive polymer actuators, ionic polymer‐metal composite (IPMC) actuators that consist of an ionic exchangeable polymer and two noble metallic electrodes, thanks to their outstanding features such as large bending strain, low driving voltage, low power consumption, and flexible biomimetic movements, have been intensively investigated as promising candidates to meet the requirements of future soft electronics.^7^, ^8^, ^9^ However, IPMC actuators have also disadvantages such as slow response time, inconsistent mechanical deformations under direct current (DC) inputs, poor responsiveness under harsh conditions, and high production cost resulting from the use of noble metal electrodes.^10^ In order to overcome these critical issues and to apply these IPMC actuators to next‐generation electronic devices, the development of more efficient and cost‐effective electrodes with best‐functioning electro‐chemomechanical properties is highly desirable for use in high‐performance electroactive artificial muscles.

Several carbon‐based electrodes including graphene,^11^ reduced graphene oxide (rGO)^12^, ^13^ and graphene papers,^14^ carbon nanotubes (CNT),^15^, ^16^ and rGO/CNT hybrid^17^ have been explored for electroactive ionic polymer actuators. Recently, the use of urea‐derived graphitic carbon nitride (g‐CN) nanosheet electrodes has been proposed for the development of high‐performance ionic actuators.^18^ Interestingly, hierarchically nanostructured porous carbons (HPC) derived from polymeric compounds, because of their high specific surface area (SSA) and impressive electrochemical functionality, have been recently introduced as electrode materials for supercapacitors, electrocatalysts, and lithium ion batteries.^19^, ^20^ Moreover, doping with heteroatoms such as nitrogen or boron has been reported to be an effective way to further enhance the electrical conductivity and electrochemical activity of HPCs.^21^, ^22^ Recent studies have revealed that hierarchically porous nitrogen‐doped carbons (HPNCs) exhibit a remarkable surface‐to‐volume ratio, with abundant nitrogen‐active sites over hierarchical pores, which are able to promote rapid electron transfer, high charge capacity, and high electrolyte accessibility.^21^, ^23^ Therefore, HPNC electrodes with a high degree of graphitic structure, hierarchical pore structures, and hetero‐atom doping are expected to contribute synergistically to outstanding electro‐chemomechanical responses in ionic‐type artificial muscles.

Microporous polymer or covalent organic frameworks have been constructed by polymerization of rigid organic building blocks with desired functional groups.^24^ Until now, covalent organic frameworks (COFs) with large specific surface area and high thermal stability have been extensively investigated for gas storage, gas separation, catalysis, and enzyme adsorption.^25^, ^26^, ^27^ Notably, the carbonization of COFs as nitrogen‐rich precursors, followed by additional postmodification using different porogens, has been considered to be a facile strategy for the synthesis of functional HPNCs materials.^23^ However, this strategy suffers from some serious drawbacks such as its time consuming nature, high cost, and high energy demand. Therefore, it is very challenging to explore novel HPNCs materials via direct one‐pot carbonization of nitrogen‐containing precursors without any post‐modification. Moreover, there has yet been no report on HPNC electrodes derived from nitrogen‐rich organic frameworks as precursors by direct one‐pot carbonization. Nevertheless, we envisage that nitrogen can be doped into hierarchically porous carbon frameworks by the direct one‐pot carbonization of a nitrogen‐containing organic framework at high temperature. Up to now, to the best of our knowledge, HPNCs derived from nitrogen‐rich polymer organic frameworks have not been explored as electrodes to develop highly bendable artificial muscles.

In this study, we for the first time report a facile strategy to synthesize, by direct one‐pot carbonization without any postmodification, HPNC materials derived from poly(triazine‐triptycene) organic frameworks (PtztpOFs) as precursor and to develop an ultrasensitive electroactive artificial muscle using PtztpOF‐derived HPNC electrodes with tailored nanoporous structures and nitrogen‐doping level. The HPNCs, owing to their abundant micro‐ and mesoporosity, large specific surface area, and dominant graphitic nitrogen doping in carbon matrix, are expected to exhibit not only exceptional electrochemical activity in both aqueous and nonaqueous electrolytes, but also rapid electron transfer through their well‐interconnected 3D hierarchical structures. To utilize the HPNC as a highly capacitive and conductive electrode in ionic polymer actuators, a conducting polymer, poly(3,4 ethylenedioxythiophene) polystyrene sulfonate (PEDOT:PSS), was introduced as a binder. An electroactive actuator that exhibits well‐functioning bending deformation even under low voltage below 0.5 V with long‐term durability was developed by depositing the HPNC/PEDOT:PSS electrode on both sides of an ionic exchangeable Nafion membrane containing ionic liquid electrolyte. Moreover, inspired by nature, we fabricated a flower‐shaped bioinspired actuating device and successfully demonstrated opening and closing motions of the device under electrical stimuli. To the best of our knowledge, HPNCs with tailored porous structure and doping level have not yet been used as electrode materials for high‐performance ionic actuators. The PtztpOF‐derived HPNC electrode is very suitable for ultrasensitive artificial muscles operating under extremely low input voltages below 1.0 V; this approach will greatly prompt technical advances in soft and flexible actuators that have been considered as a key component in next‐generation electronic products.

## 2 Results and Discussion

**Figure 1:**
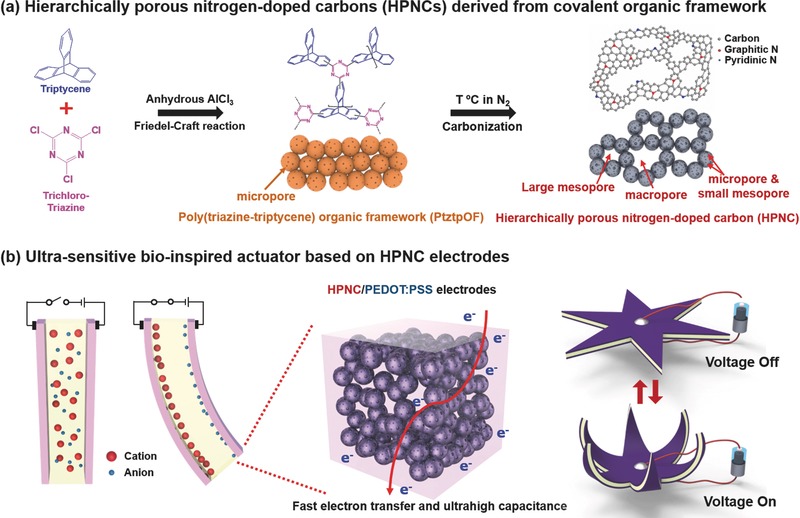
Schematic diagrams for synthetic route of PtztpOF and HPNCs and the concept of a novel ionic artificial muscle using HPNCs as electrode materials. a) Scheme of synthesis of PtztpOF and HPNC materials from PtztpOF precursor. The direct one‐pot carbonization of a PtztpOF precursor at 900 °C results in the formation of HPNC‐900 material with hierarchical porosity including both micro‐, meso‐, and marcoporosity. b) Bioinspired ionic artificial muscles with HPNC‐900/PEDOT:PSS electrodes. Hierarchical porous structures of HPNC‐900 in PEDOT:PSS matrix lead to fast electron transfer and rapid ion seperation, resulting in large bending performance of the actuator. A bioinspired flower‐like actuating device was successfully demonstrated.

The syntheses of PtztpOF and HPNC materials derived from the PtztpOF precursor are shown in **Figure** **1**a. The synthesis of the PtztpOF was conducted by Friedel−Crafts condensation of trichloro‐triazine and triptycene in the presence of anhydrous AlCl_3_ as catalysts in dichloromethane. The N_2_ adsorption‐desorption isotherm of PtztpOF exhibits type‐I features associated with sharp uptake in the low‐pressure region and the corresponding pore size distribution (PSD) plot shows a maximum value at around 1.30 nm, suggesting that micropores are dominant in PtztpOF (Figure S1a,b, Supporting Information). The combination of high BET surface area (392.88 m^2^ g^−1^) with microporosity and large nitrogen content makes the PtztpOF precursor an attractive candidate as a carbon source to prepare HPNC‐based electrode materials via direct one‐pot carbonization. Therefore, the direct one‐pot carbonization of a PtztpOF precursor was carried out in a ceramic boat, which was placed in a furnace at two different temperatures of 700 and 900 °C under N_2_ flow. The HPNCs obtained at 700 and 900 °C are denoted as HPNC‐700 and HPNC‐900, respectively. As schematically shown in Figure 1b, the novel electroactive bioinspired actuator is fabricated by drop‐casting of HPNC‐900/PEDOT:PSS on an ion‐exchangeable Nafion‐ionic liquid membrane, resulting in bending deformations under electric fields. It is noteworthy that highly interconnected HPNC structures in the PEDOT:PSS facilitate rapid electron transfer, and high electrical conductivity, electrochemical activity and mechanical property. Such high electro‐chemomechanical property of HPNC‐900/PEDOT:PSS electrodes can lead to fast ion separations in the Nafion membrane under electrical stimuli. As a result, the fabricated electroactive bioinspired actuator can exhibit large bending deformation under ultralow input voltage, without significant performance degradation for long‐term operations. In addition, in order to get closer to practical applications, an actuating device inspired by nature is designed, as shown in Figure 1b.

**Figure 2:**
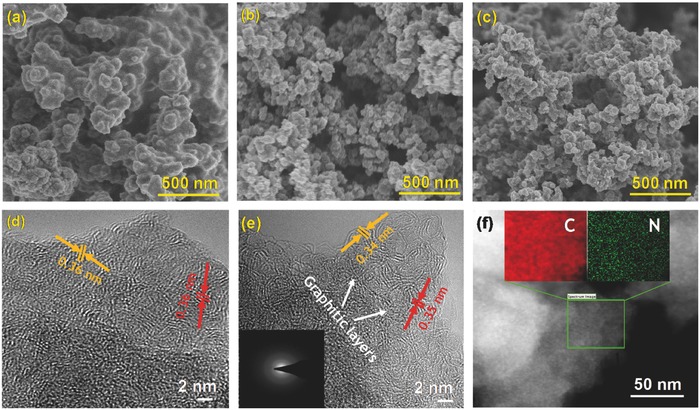
Morphological analyses of HPNC materials. FESEM images of a) PtztpOF, b) HPNC‐700, and c) HPNC‐900. High‐resolution TEM image of d) HPNC‐700 and e) HPNC‐900; inset showing corresponding SAED pattern of HPNC‐900. f) STEM image of HPNC‐900 with corresponding Carbon and Nitrogen elemental mapping images.

Field emission scanning electron microscopy (FESEM) and high‐resolution transmission electron microscopy (HRTEM) images were employed to investigate the structural morphologies of the PtztpOF and HPNC materials, as shown in **Figure** **2**. Figure 2a demonstrates that the as‐synthesized PtztpOF sample consists of interlinked irregular spherical particles exhibiting relatively smooth surfaces with diameters in a range of 100–200 nm. Notably, after carbonization at high temperatures, the FESEM images of HPNC‐700 and HPNC‐900 reveal the formation of sphere‐like aggregates of carbon nanoparticles with a diameter range of 40–80 nm (Figures 2b and 2c). These aggregates make 3D continuous porous carbon nanonetworks by interconnecting in different directions. Importantly, surface roughness of the HPNC particles gradually increased with carbonization temperature, implying the formation of micropores and small‐sized mesopores within carbon nanoparticles. Moreover, the compact and loose aggregation of carbon particles generated largely interconnected mesopores and macropores throughout the carbon nano‐network in both HPNC‐700 and HPNC‐900, confirming the hierarchically porous structures. Further, high‐resolution TEM image of HPNC‐900 reveals very clear graphitic layers with a spacing of around 0.34–0.35 nm, which is slightly lower than that of HPNC‐700 (spacing of about 0.36 nm), as depicted in Figure 2d,e. These findings imply that HPNC‐900 has a higher degree of graphitic structure than HPNC‐700 in accordance with PXRD, Raman, and XPS results. The doping of nitrogen in HPNC‐900 was further confirmed by the elemental mapping images as shown in Figure 2f. It reveals that nitrogen atoms are homogeneously distributed throughout the HPNC nanonetworks.

**Figure 3:**
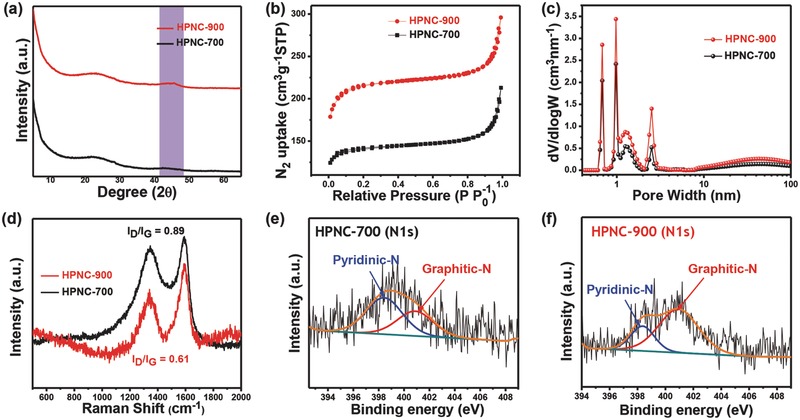
Characterization of PtztpOF and HPNCs. a) Powder X‐ray diffraction (PXRD) patterns of HPNC‐700 and HPNC‐900. b) N_2_ adsorption–desorption isotherms and c) corresponding pore‐size distributions of HPNC‐700 and HPNC‐900. d) Raman spectra of HPNC‐700 and HPNC‐900. High‐resolution XPS spectra of N1s for e) HPNC‐700 and f) HPNC‐900.

The formation of PtztpOF was confirmed by solid‐state ^13^C cross‐polarization magic angle spinning (CP‐MAS) nuclear magnetic resonance (NMR) and Fourier transform infrared spectroscopy (FTIR) results, as described in the Supporting Information (Figure S2a,b). Interestingly after carbonization at varying temperatures, some fine FTIR bands of the original PtztpOF disappear in the HPNC materials (Figure S2b, Supporting Information). The FTIR spectra of HPNC‐700 and HPNC‐900 provide clear evidence for the presence of surface functional groups originating from N and O heteroatoms, as illustrated in Supporting Information. Furthermore, the powder X‐ray diffraction (PXRD) patterns of HPNC‐700 and HPNC‐900 are shown in **Figure** **3**a; these patterns show two weak diffraction peaks located at around 23.5° and 43.5° corresponding to the (002) and (101) planes of graphitic carbon materials, respectively.^28^ In comparison with HPNC‐700, HPNC‐900 was found to exhibit higher intensity of the 2θ peak at around 43.5° due to the formation of a higher degree of graphitic structure, which greatly improves the electrical conductivity and electrochemical performance.^28^, ^29^ It is well known that the capacitive performance of HPNC‐based electrodes depends on the SSA, pore sizes and pore structures of HPNCs.^30^ Therefore, to further investigate the SSA and porosity of HPNC‐700 and HPNC‐900, N_2_ sorption isotherms and the PSD were analyzed by Brunauer–Emmett–Teller (BET) and nonlocalized density functional theory (NLDFT) measurements, respectively (Figure 3b,c). In Figure 3b, typical type II isotherms were obtained for both HPNC‐700 and HPNC‐900. Especially, the N_2_ uptake at relatively low pressure (*P*/*P*_0_ < 0.2) implies that the existence of abundant micropores in the HPNCs and in the intermediate region of *P*/*P*_0_ = 0.2–0.8 suggests the relatively low content of small‐sized mesopores. Further, at relatively higher pressures approaching 1, sharp increases in the amount of N_2_ uptake were observed for both of these HPNCs materials, implying the existence of large mesopores or macropores.^31^ It is noted that with the increase in carbonization temperature, the SSA and pore volume increases from 631.49 to 830.46 m^2^ g^−1^ and from 0.37 to 0.45 cm^3^ gm^−1^ for HPNC‐700 and HPNC‐900, respectively; which implies the increase of micropores in the carbon nanonetworks. The PSD curves for both HPNC materials reveal that micropores and small‐sized mesopores within the carbon nanoparticles exhibited at 0.68, 0.97, and 2.54 nm, while relatively large mesopores or macropores among the carbon nanonetwork are basically concentrated in a size range from 20 to 65 nm, confirming the hierarchical pore structure of the HPNCs materials (Figure 3c). Interestingly, the PSD peak intensities for the corresponding micropores, as well as for the mesopores in HPNC‐900, are comparatively higher than those for HPNC‐700, implying that all types of pores become more abundant at higher carbonization temperature.^32^, ^33^ Based on the above findings, HPNC‐900, with highly tailored hierarchical porous structures, high SSA and large pore volume, is expected to be an excellent electrode candidate with superior electrochemical properties necessary for high‐performance ionic actuators. The Raman spectra of HPNC‐700 and HPNC‐900 (Figure 3d) present the D‐bands at around 1345 cm^−1^ associated with a disordered carbon structure and the G‐bands at around 1585 cm^−1^ related to a graphitic carbon structure, without significant peak shift.^28^ Importantly, the *I*_D_/*I*_G_ ratio of HPNC‐700 (≈0.89) is higher compared to HPNC‐900 (≈0.69) revealing the formation of higher degree of graphitic structure in HPNC‐900, which is in good agreement with the PXRD patterns.^28^ X‐ray photoelectron spectra (XPS) analysis was further conducted to estimate the chemical binding state and quantitative elemental composition of HPNC‐700 and HPNC‐900 materials (Figure 3e,f and Figure S2c–f, Supporting Information). The XPS survey spectra indicates that the successful incorporation of N atoms into the as‐prepared HPNCs materials by the direct one‐pot carbonization of PtztpOF (Figure S2c, Supporting Information). The residual nitrogen content (1.94% atomic percent) in HPNC‐900 is lower than in HPNC‐700 (2.71%) due to the higher carbonization temperature (Table S1, Supporting Information).The high‐resolution deconvoluted N 1s spectra for HPNC‐700 and HPNC‐900 indicate the existence of pyridinic nitrogen at ∼398.5 eV and graphitic nitrogen at ≈401.1 eV, respectively, as shown in Figure 3e,f. Notably, the relative content of graphitic N, evaluated from the N 1s deconvoluted spectra, is more abundant in HPNC‐900 (70%) than in HPNC‐700 (38%) (Figure S2d, Supporting Information), indicating that nitrogen atoms mainly reside in the graphitic layers instead of at the periphery, which can significantly increase the electrical conductivity as well as the electrochemical response of HPNC‐900 compared to those characteristics of HPNC‐700.^34^ Therefore, HPNC‐900 can be a highly desirable electrode material for high‐performance ionic actuators.

**Figure 4:**
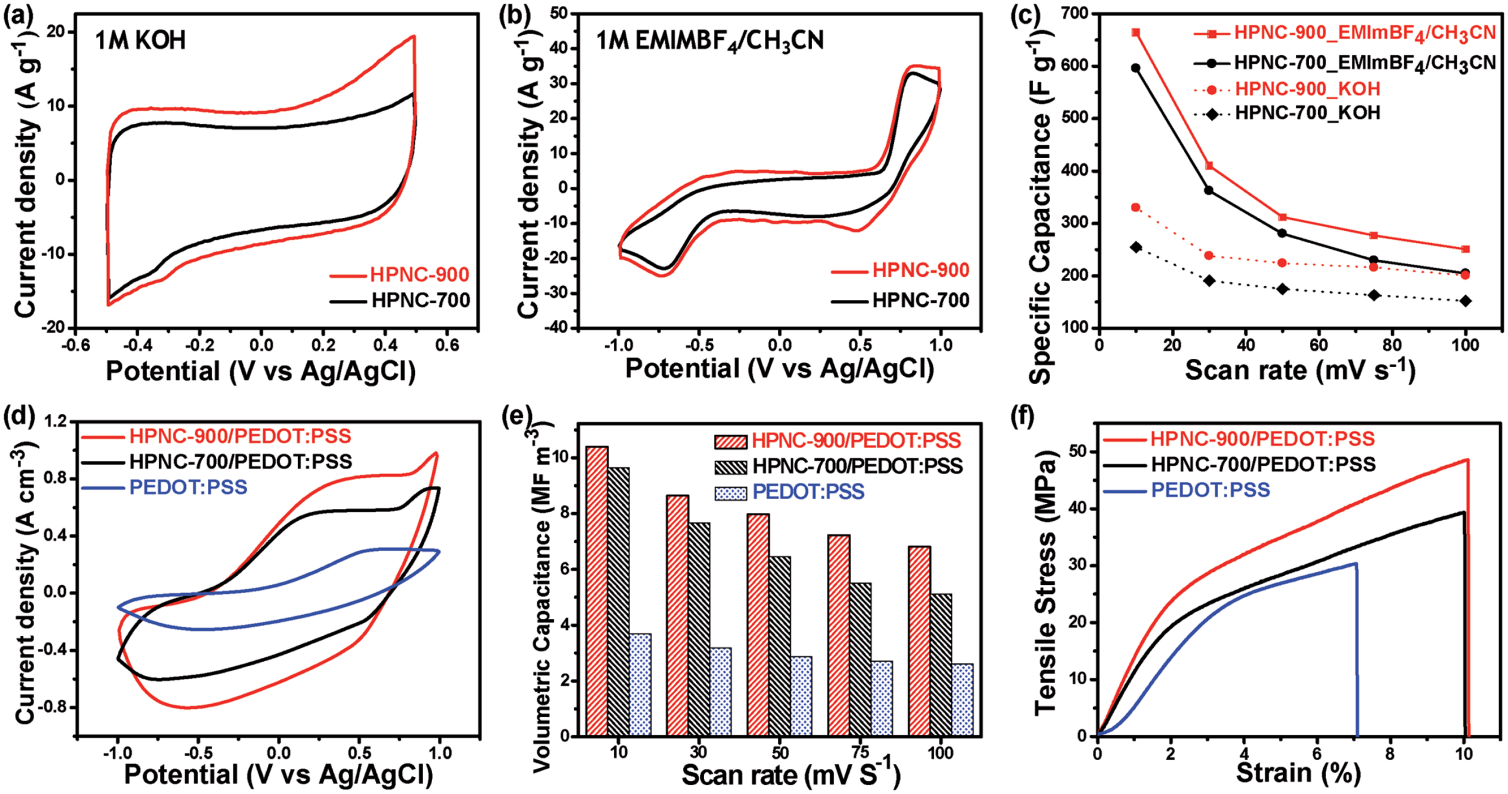
Electrochemical and mechanical performances of HPNC based electrodes. Cyclic voltammetry analysis of HPNC‐700 and HPNC‐900 in a) 1.0 m KOH and b) 1.0 m EMIM‐BF_4_/CH_3_CN electrolytes at a scan rate of 100 mV s^−1^. c) Specific capacitance values of HPNC‐700 and HPNC‐900 at various scan rates. d) Cyclic voltammetry analysis of PEDOT:PSS, HPNC‐700/PEDOT:PSS, and HPNC‐900/ PEDOT:PSS electrodes in a 1 m EMIM‐BF_4_/CH_3_CN electrolyte at a scan rate of 100 mV s^−1^. e) Volumetric capacitance of the electrodes at various scan rates. f) Stress–strain curves of the electrodes.

To explore actuation performance, the electrochemical properties of the obtained HPNC materials and composite electrodes were investigated using cyclic voltammetry (CV) tests in both 1 m KOH and 1 m EMIM‐BF_4_/CH_3_CN electrolytes (**Figure** **4**a,b). Figure 4a presents the CV responses of HPNC‐700 and HPNC‐900 in a 1 m KOH solution at a scan rate of 100 mV s^−1^ in the potential window of +0.5 to −0.5 V. The CV curves of all the HPNC materials exhibit a rectangular‐like shape, indicating close to ideal electrical double‐layer capacitor (EDLC) behavior. Moreover, the rectangular shapes of all the HPNC materials are well maintained at various scan rates in a range of 10–100 mV s^−1^, suggesting the excellent electrochemical performance of these HPNCs as electrode materials (Figure S3a,b, Supporting Information). Figure 4c shows the scan rate dependency of the specific capacitance for the HPNC materials in both aqueous and nonaqueous electrolytes. HPNC‐900 exhibited an appreciably higher specific capacitance than that of HPNC‐700 at all scan rates. Significantly, HPNC‐900 exhibited a higher specific capacitance (330 F g^−1^) than that of HPNC‐700 (254 F g^−1^) at a scan rate of 10 mV s^−1^ in a 1 m KOH solution. Notably, the specific capacitance of HPNC‐900 was higher than those of previously reported N‐doped graphene and N‐doped carbon materials.^13^, ^18^, ^19^, ^21^, ^23^, ^30^, ^34^ Furthermore, the CV responses of HPNC‐700 and HPNC‐900 at a scan rate of 100 mV s^−1^ were recorded in nonaqueous electrolyte (1 m EMIM‐BF_4_/CH_3_CN), since our dry‐type air‐working actuator will be operated with a nonaqueous ionic liquid, EMIM‐BF_4_ (Figure 4b and Figure S3c,d, Supporting Information). Obviously, slight distortion in the rectangular CV curves of both electrode materials occurs in ionic liquid medium, which might be due to the strong ionic interactions between the IL, EMIM‐BF_4_ and the HPNC frameworks.^35^ Finally, we inferred that HPNC‐900 exhibits noticeably higher specific capacitances in both aqueous and nonaqueous electrolytes than the HPNC‐700 electrode, as shown in Figure 4c. The superior specific capacitance of HPNC‐900 is ascribed to its highly developed hierarchically porous structures, with abundant micro‐ and mesopores, high specific surface area, and high content of graphitic nitrogen doping. In general, micropores can enlarge the SSA to improve the EDLC via efficient accumulation of the electrolyte on the electrode surface, while mesopores facilitate rapid ion diffusion by serving as low‐resistance pathways.^23^ Additionally, the presence of rich graphitic nitrogen in the carbon matrix is beneficial for a higher conductivity.^34^ Hence, HPNC‐900 herein is the most promising electrode material for the exploration of efficient ultralow‐voltage‐driven ionic actuators. Moreover, the CV responses of HPNC materials and the cyclic stability of HPNC‐900 were also analyzed in only ionic liquid‐electrolyte medium as shown in Figure S4 of the Supporting Information. Very small redox peaks were found to appear at all scan rates from 10 to 100 mV s^−1^ in EMIM‐BF_4_ medium in comparison with those of the CV responses in 1 m EMIM‐BF_4_/CH_3_CN electrolyte medium. Therefore, the actuation performance and their durability of the ionic actuators were carried out by employing EMIM‐BF_4_ embedded Nafion based ionic membrane. Additionally, we also explored the CV performances of PEDOT:PSS, HPNC‐700/PEDOT:PSS, and HPNC‐900/PEDOT:PSS electrodes to evaluate the effects of charge transports of each electrodes in nonaqueous electrolyte. Figure 4d provides the CV curves of all the electrodes at a scan rate of 100 mV s^−1^ in the potential window of −1.0 to +1.0 V. Besides this, the CV responses of all the electrodes at varying scan rates (10–100 mV s^−1^) are shown in Figure S5a–c (Supporting Information). The nature of the CV curve for the PEDOT:PSS electrode is quite similar to that in our previous reports.^13^ The volumetric capacitance of the HPNC‐900/PEDOT:PSS electrode was found to exhibit highest value (10.402 MF m^−3^) at a scan rate of 10 mV s^−1^ compared to the values of the HPNC‐700/PEDOT:PSS (9.646 MF m^−3^) and PEDOT:PSS (3.760 MF m^−3^) electrodes, as displayed in Figure 4e. Remarkably, the volumetric capacitance of the HPNC‐900/PEDOT:PSS electrode was found to exhibit a value of 6.820 MF m^−3^ even at a high scan rate of 100 mV s^−1^, suggesting good rate capability of this flexible electrode. Importantly, the excellent CV response for the HPNC‐900/PEDOT:PSS electrode is mainly attributed to the combined contribution of the EDL capacitance of HPNC‐900 and the high conductivity of PEDOT:PSS. An electrode with such excellent electrochemical performance is expected to generate large bending displacement of the corresponding ionic actuator. We further investigated the stress–strain mechanical responses of the electrodes, which can influence the bending performance of the ionic actuators. Figure 4f and Table S2 (Supporting Information) show the reinforcing effects of the HPNC‐700/PEDOT:PSS and HPNC‐900/PEDOT:PSS electrodes, respectively, compared with the pristine PEDOT:PSS electrode. The tensile strength of the HPNC‐900/PEDOT:PSS electrode (48.56 MPa) is much higher than that of the HPNC‐700/PEDOT:PSS (39.36 MPa) and PEDOT:PSS (30.26 MPa) electrodes. Similarly, the HPNC‐900/PEDOT:PSS electrode exhibited a higher tensile modulus (1.46 GPa) than that of both HPNC‐700/PEDOT:PSS (1.14 GPa) and pristine PEDOT:PSS (0.86 GPa) electrodes. These significant improvements in the tensile strength and modulus of the HPNC‐900/PEDOT:PSS electrode are attributed to the synergistic contribution of HPNC‐900 and PEDOT:PSS. The hierarchical micro‐ and mesoporosity and larger specific surface area of HPNC‐900 in comparison with those characteristics of HPNC‐700 facilitate a stronger interfacial interaction with the PEDOT:PSS matrix, eventually resulting in higher mechanical properties. Appreciably, both the HPNC‐700/PEDOT:PSS and HPNC‐900/PEDOT:PSS electrodes were found to exhibit higher elongation at break than that of the pristine PEDOT:PSS. In this regard, the notable enhancement of the mechanical properties is attributed to the higher surface area and the nitrogen‐doping of HPNCs, which offer promising interfacial interactions with PEDOT:PSS. Furthermore, the electrical conductivities of the HPNC‐700/PEDOT:PSS, HPNC‐900/PEDOT:PSS, and PEDOT:PSS electrodes were also measured using a four probe method. The conductivity of the HPNC‐900/PEDOT:PSS electrode (0.073 MS m^−1^) is much higher than that of HPNC‐700/PEDOT:PSS (0.058 MS m^−1^) and PEDOT:PSS (0.036 MS m^−1^) electrodes, as shown in Table S2 (Supporting Information). Especially, the ameliorated conductivity of the HPNC‐900/PEDOT:PSS electrode is ascribed to the existence of a higher degree of graphitic structure, along with abundant of graphitic nitrogen in the carbon nanonetworks of HPNC‐900, which can facilitate stronger interactions with adjacent sulfonate groups of PEDOT:PSS (Figure S6, Supporting Information). In addition, the existence of pyridinic N in HPNC‐900 also provides strong interfacial interaction with sulfonic acid groups of PEDOT:PSS through H‐bonding, resulting in better compatibility (Figure S6, Supporting Information).

**Figure 5:**
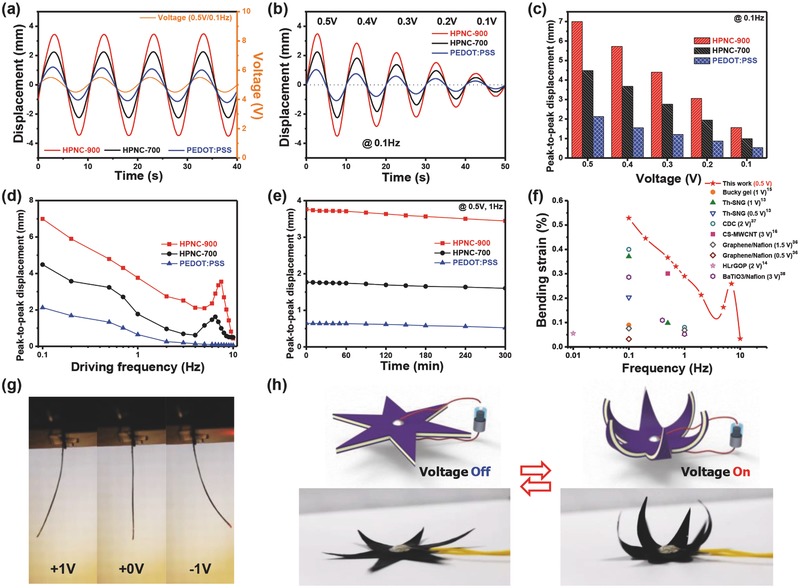
Bending actuation performances of ultralow‐voltage‐driven ionic actuators. a) Bending actuation performances of cantilevered ionic actuators based on PEDOT:PSS, HPNC‐700/PEDOT:PSS, and HPNC‐900/PEDOT:PSS electrodes under harmonic excitation of 0.5 V at 0.1 Hz. b) Bending performances of the electrodes under various peak voltages (0.1–0.5 V) at 0.1 Hz. c) Peak‐to‐peak displacement of the actuators at different input voltages of 0.1–0.5 V. d) Peak‐to‐peak displacement of the actuators under different applied frequencies. e) Time‐dependent displacement of actuators under excitation of 0.5 V at 0.1 Hz, showing high durability for 5 h. f) Bending strain‐frequency curve of the actuator in comparison with that of other soft ionic type actuators reported in the literature with a focus on low‐voltage operation. g) Optical images of the symmetrically bent actuators under input voltage of ±1 V at excitation frequency of 0.1 Hz. h) Bioinspired flower‐shaped actuating device responding to ultralow electrical stimulus.

The actuation performances of electroactive artificial muscles sandwiched with HPNC/PEDOT:PSS composite electrodes were investigated under various electric inputs, as shown in **Figure** **5**. EMIM‐BF_4_, one of the hydrophilic ionic liquids, was embedded in Nafion to provide Nafion/EMIM‐BF_4_ as ionic composite membrane for artificial muscles. As shown in Figure S7 (Supporting Information), ionic interactions between hydrophilic EMIM‐BF_4_ and sulfonic acid groups of Nafion lead to fast ion transports. In addition, the cross‐section image of the as‐tested artificial muscle is presented in Figure S8 (Supporting Information). Figure 5a presents the bending deformation responses of three different ionic actuators having pure PEDOT:PSS, HPNC‐700/PEDOT:PSS, and HPNC‐900/PEDOT:PSS electrodes under an ultralow peak voltage of ±0.5 V in a sinusoidal electrical input with a period of 10 s. The HPNC‐900/PEDOT:PSS‐based actuator exhibited an exceptionally large bending displacement of 6.99 mm only under the peak voltage of 0.5 V, which is 3.30 and 1.56 times higher than that of PEDOT:PSS‐based and HPNC‐700/PEDOT:PSS‐based actuators, respectively. The high bending actuation performance of the HPNC‐900/PEDOT:PSS‐based actuator under ultralow input voltages is mainly attributed to the interconnected hierarchically porous carbon structures, higher graphitic N content and superior mechanical and electrochemical properties of the HPNC‐900/PEDOT:PSS electrode, resulting in efficient electron and ion pathways. Because the electron pathways facilitate fast electron transfer throughout the whole electrode, dissociated cations and anions of ionic liquids inside the ionic polymer are attracted to the electrodes in a short time, resulting in large bending actuation and fast movements. In other words, the results are highly correlated to the excellent electro‐double‐layer capacitance performance of HPNC‐900/PEDOT:PSS, previously shown in Figure 4d,e. Moreover, to verify the performance of HPNC‐900/PEDOT:PSS‐based actuator at lower driving voltages, the bending displacement of the actuator was evaluated under ultralow input voltages of ±0.1, ±0.2, ±0.3, ±0.4, and ±0.5 V at a cycle time of 10 s, as shown in Figure 5b. The tip displacements of the actuators were found to decrease almost linearly with the decreasing input voltages. Under ultralow input voltages, the HPNC‐900/PEDOT:PSS‐based actuator exhibits exceptionally large bending deformations in comparison with those of the other two actuators. Interestingly, under an ultralow input voltage of 0.1 V at 0.1 Hz, the tip displacement of the HPNC‐900/PEDOT:PSS‐based actuator reached to 1.56 mm, about 2.94 and 1.58 times higher than those values of the other actuators, as shown in Figure 5c. This result indicates that the HPNC‐900/PEDOT:PSS‐based actuator can successfully work under extremely low input voltages. To the best of our knowledge, such levels of actuation performances have not been reported until now in the field of electroactive ionic polymer actuators. Furthermore, we evaluated the peak‐to‐peak displacement of the HPNC‐900/PEDOT:PSS‐based actuator under the excitation voltage of ±0.5 V at nine different applied frequencies, as depicted in Figure 5d. The HPNC‐900/PEDOT:PSS‐based actuator shows much larger bending deformation with the decreasing of applied frequency. A 5 h durability test was also conducted to verify the sustainability of the HPNC‐900/PEDOT:PSS‐based actuator, as presented in Figure 5e. The HPNC‐900/PEDOT:PSS‐based actuator shows a remarkable durability due to the high mechanical and electrochemical properties of the HPNC‐900/PEDOT:PSS electrodes. Figure 5f shows the bending strain‐frequency curve of the HPNC‐900/PEDOT:PSS‐based actuator in comparison with those of other ionic type actuators published in the literature, which have focused on low‐voltage operation.^13^, ^14^, ^15^, ^16^, ^36^, ^37^, ^38^ As shown in Figure 5f, the HPNC‐900/PEDOT:PSS‐based actuator, operated under an ultralow peak voltage of ±0.5 V, outperforms the reported ionic type actuators in regard to bending strain. The electromechanical energy efficiencies of the actuators were investigated and are shown in Figure S9 and Table S3 (Supporting Information). The HPNC‐900/PEDOT:PSS‐based actuator exhibits 1.98 times and 1.79 times higher energy efficiency than that of the PEDOT:PSS or the HPNC‐700/PEDOT:PSS‐based actuators, respectively; this HPNC‐900/PEDOT:PSS‐based actuator also has outstanding energy conversion capability to transform electrical energy into mechanical kinetic energy. Moreover, the blocking forces of the actuators were examined under the DC input of 2 V as shown in Figure S10 (Supporting Information). Owing to its high mechanical stability and efficient energy conversion capability, the HPNC‐900/PEDOT:PSS‐based actuator generated a blocking force of 0.998 mN, which is 1.37 and 2.34 times larger than the other two actuators, respectively. In addition, in order to clearly compare with other previous actuators, the blocking forces of the actuators were normalized to the dimensions of the each actuator.^39^ The normalized blocking forces of PEDOT:PSS, HPNC‐700/PEDOT:PSS, and HPNC‐900/PEDOT:PSS are 0.227, 0.389, and 0.532 MPa, respectively. Optical images of the symmetrically bent actuators under input voltage of ±1 V at excitation frequency of 0.1 Hz are displayed in Figure 5g. Particularly, in order to demonstrate the soft robotic capability of the actuator, a flower‐shaped actuating device that responds to ultralow electrical stimulus was fabricated by mimicking a type of flowers that can often be encountered in nature, as presented in Figure 5h and Video S1 (Supporting Information). When an electric signal was applied, such a nature‐inspired actuating device showed a closed bending shape, causing it to enfold an object in a manner similar to that of the flower Dionaea muscipula.

## 3 Conclusions

In summary, we report that HPNCs derived from a microporous poly(triazine‐triptycene) organic framework can serve as superb electrode materials for ultrasensitive bioinspired artificial muscles operated under ultralow electric fields. HPNC‐900, synthesized by facile one‐pot carbonization of poly(triazine‐triptycene) organic frameworks, was found to exhibit high specific capacitance of 330 F g^−1^ due to its large specific surface area (830.46 m^2^ g^−1^), hierarchical micro‐ and mesoporous structures. Graphitic nitrogen doping into the carbon matrix at higher carbonization temperature was also introduced to obtain improved electrical and electrochemical properties of HPNCs. Furthermore, the hierarchically micro‐ and mesoporosity, the large specific surface area, and nitrogen‐doping of the HPNCs play key roles in providing strong interaction between PEDOT:PSS and HPNCs, resulting in a much higher electro‐chemomechanical property of the HPNC/PEDOT:PSS electrodes. Especially, the HPNC‐900/PEDOT:PSS electrodes, which exhibited high volumetric capacitance of 10.402 MF m^−3^, electrical conductivity of 0.073 MS m^−1^, and tensile modulus of 1.46 GPa, were utilized for ultralow voltage‐operable ionic actuators. The electroionic antagonistic muscles based on HPNC‐900/PEDOT:PSS electrodes demonstrated remarkable actuation performance at ultralow input voltage ±0.5 V, including large bending deformation of 6.99 mm and 5 h durability. Therefore, we anticipate that this approach will provide a new direction for developing the biomimetic soft actuators needed for next‐generation flexible and wearable electronics. Also, based upon our study, a Dionaea muscipula‐inspired flower device successfully demonstrated under the controlled electric signals.

## 4 Experimental Section

*Synthesis of PtztpOF*: The PtztpOF precursor was synthesized by Friedel−Crafts condensation of 2,4,6‐trichloro‐1,3,5‐triazine and triptycene. In a typical synthesis, a 100 mL round‐bottomed flask was charged with triptycene (257 mg, 1.0 mmol), 2,4,6‐trichloro‐1,3,5‐triazine (184 mg, 1.0 mmol), and anhydrous AlCl_3_ (400 mg, 3 mmol) under N_2_ atmosphere. The mixture was refluxed in 60 mL of dichloromethane for 20 h. After cooling, an orange‐colored solid was obtained by filtration. Then, the solid was washed with deionized water (50 mL), MeOH (50 mL), THF (50 mL), and acetone (50 mL), and dried at 120 °C under vacuum for 24 h to obtain the orange‐colored powder form of PtztpOF. The yield was about 95%.

*Preparation of Hierarchical Porous Nitrogen‐Doped Carbons (HPNC‐700 and HPNC‐900) from the PtztpOF Precursor*: The as‐prepared PtztpOF precursor was transformed into HPNCs by calcination at two different temperatures (700 and 900 °C) under an N_2_ atmosphere at a heating rate of 5 °C min^−1^ in a tube furnace, and kept at this temperature for 2 h. After cooling to room temperature, the as‐formed black powders were washed several times with water and ethanol, and then dried at 60 °C overnight. Then, resultant materials, denoted as HPNC‐700 and HPNC‐900, were easily obtained.

*Fabrication of Flexible Composite Electrodes, HPNC‐700/PEDOT:PSS and HPNC‐900/PEDOT:PSS*: The aqueous PEDOT:PSS solution containing 5 vol% of DMF, 0.25 mg mL^−1^ of HPNC‐900 was stirred for 12 h at ambient temperature to obtain a homogeneous mixture. The resulting mixture was then poured into a glass Petri dish and dried at 50 °C for 16 h. A flexible composite electrode of HPNC‐700/PEDOT:PSS was prepared in a similar way. Further, the pristine PEDOT:PSS film was also prepared in the same way without adding HPNC materials.

*Fabrication of Ionic Artificial Muscles*: Using a dropper, layer‐by‐layer sandwiched ionic actuators were fabricated by direct deposition of the mixtures of HPNC‐900/PEDOT:PSS (or HPNC‐700/PEDOT:PSS) with DMF or of the solution of PEDOT:PSS with DMF as electrodes on both sides of a Nafion‐EMIM‐BF_4_ composite membrane; mixtures were then dried at 120 °C for 30 min. The multilayered ionic artificial muscles having these electrode materials were successfully fabricated with structural integrity and strong bonding between Nafion and HPNC/PEDOT:PSS. All actuator samples had the same geometry of 20 mm × 3 mm × 100 µm (length × width × thickness) with a free length of 16 mm in a cantilever model.

*Materials Characterization*: The solid‐state CP/MAS (cross‐polarization with magic angle spinning) ^13^C NMR spectrum was recorded on a Bruker Avance 400 WB spectrometer at 100.61 MHz. FTIR spectra were collected on aNicolet iS50 FTIR spectrometer in the region of 400−4000 cm^−1^, using a KBr pellet method. The PXRD patterns were measured using a SmartLab X‐ray diffractometer with Cu Kα radiation. Further, all of the samples were characterized by HRTEM (Tiatan cubed G2 60–300 at 80 kV), FESEM (Magellan400), XPS (Thermo VG Scientific Sigma Probe equipped with monochromatic Al Kα‐source), and Raman spectra (ARAMIS Horiba JobinYvon). To obtain the specific surface area (SSA), total pore volume, and pore size distribution (PSD) of the samples, N_2_ adsorption−desorption measurements were performed at 77 K with a BELSORP‐max surface area analyzer.

*Electrochemical, Electrical, Mechanical, and Actuation Measurements*: CV of the HPNC‐based electrodes was performed on a multichannel potentiostat/galvanostat (VersaStat, Princeton Applied Research) in a potential range of −0.5 to +0.5 in 1 m KOH and in a potential range of –1 to +1 V in 1 m EMIM‐BF_4_ at a scan rate of 10‒100 mV s^−1^. The CV measurement was conducted using a three‐electrode system that included an active material (HPNC)‐loaded working electrode, a platinum wire as a counter electrode, and Ag/AgCl in 3.5 m KCl as the reference electrode. The specific capacitance or volumetric capacitance (F g^−1^ or F cm^−3^) in CV measurements was calculated using the following equation

(1) $$C_{\mathrm{sp}}=\frac{1}{\Delta V.\;v.\;S}\int_{V_{1}}^{V_{2}}IdV$$

where Δ*V*, *v*, and *S* are the potential window, scan rate, and weight or volume of the active materials. The sheet resistances of the flexible electrodes were measured with a probe station (MSTECH, model 4000). Further, the mechanical properties of all the electrodes were measured using a table‐top universal testing machine (AGS‐X, Shimadzu Corp.). Furthermore, a current amplifier (UPM1504) equipped with an NI‐PXI data acquisition system (NI‐PXI 1042Q, PXI 6252 board) was used to activate the actuators by generating electrical input signals. The bending performance of the actuators was evaluated using a laser displacement sensor (Keyence, LK‐031). The blocking force of the actuators was measured using a load cell (LVS‐5GA, Kyowa). The photographs of the setup are shown in Figure S11 (Supporting Information). The strain of the actuator was estimated using the following equation

(2) $$\varepsilon =\frac{2\delta d}{l^{2}+\;\delta^{2}}$$

where *l*, δ, and *d* are the free length, bending displacement, and thickness of the actuators, respectively.

## Conflict of Interest

The authors declare no conflict of interest.

## Supporting information

Supplementary

Supplementary
