# Supplementary material for: Electroionic Antagonistic Muscles Based on Nitrogen‐Doped Carbons Derived from Poly(Triazine‐Triptycene)
Source: Adv Sci (Weinh). 2017 Oct 11;4(12):1700410. doi: 10.1002/advs.201700410 (PMC5737102; doi:10.1002/advs.201700410)
Supplement: Supplementary file 1 — Supplementary [file ADVS-4-na-s001.pdf]

Copyright WILEY-VCH Verlag GmbH & Co. KGaA, 69469 Weinheim, Germany, 2017.

Supporting Information

for *Adv. Sci.,* DOI: 10.1002/advs.201700410

Electroionic Antagonistic Muscles Based on Nitrogen-Doped Carbons Derived from Poly(Triazine-Triptycene)

*Sandipan Roy, Jaehwan Kim, Moumita Kotal, Kwang Jin Kim, and Il-Kwon Oh**

Copyright WILEY-VCH Verlag GmbH & Co. KGaA, 69469 Weinheim, Germany, 2013.

Supporting Information

Electro-Ionic Antagonistic Muscles Based on Nitrogen-Doped Carbons Derived from Poly(triazine-triptycene)

Sandipan Roy[#], *Jaehwan Kim*[#], *Moumita Kotal, Kwang Jin Kim and Il-Kwon Oh*[*]

Prof. I.-K. Oh, Dr. S. Roy, J. Kim & Dr. M. Kotal
Creative Research Initiative Center for Functionally Antagonistic Nano-Engineering, Department of Mechanical Engineering, Korea Advanced Institute of Science and Technology (KAIST), 291 Daehak-ro, Yuseong-gu, Daejeon 34141, Republic of Korea

Prof. K.J. Kim
Active Materials and Smart Living Laboratory, Department of Mechanical Engineering, University of Nevada, Las Vegas (UNLV), NV 89154, USA

Microporous nature of PtztpOF was clearly revealed by N_2 adsorption-desorption isotherm measurement, as displayed in Figure S1a. In Figure S1a, the isotherm of PtztpOF shows a rapid nitrogen uptake in the low-pressure region ($P/P_0 < 0.02$), implying that micropores are prominent in the sample.[1-3] PtztpOF shows a specific surface area (BET) of 392.88 m^2 g^{-1} and a total pore volume of 0.33 cm^3 gm^{-1}. Further, the pore size distribution (NLDFT) of PtztpOF exhibits a maximum at 1.30 nm, suggesting an abundance of micropores (Figure S1b).

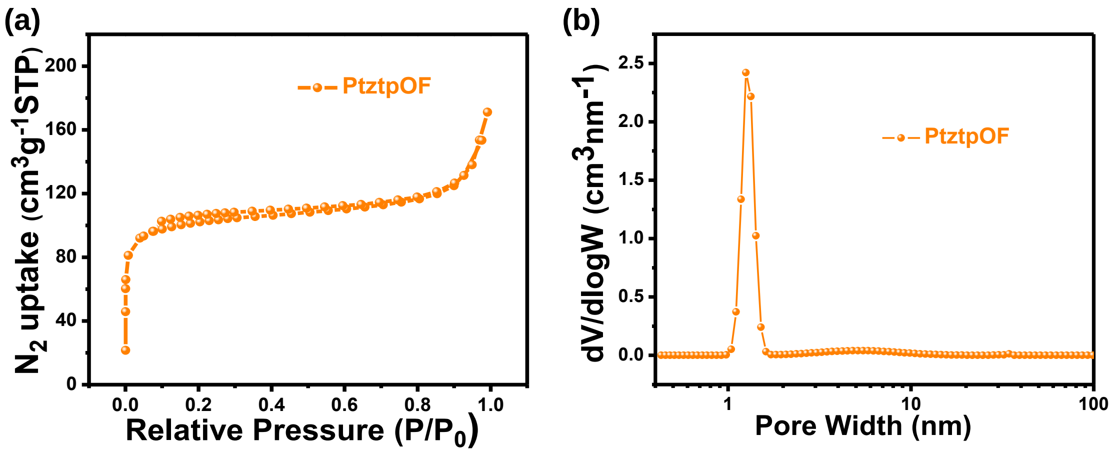

Figure S1. (a) Nitrogen adsorption-desorption isotherm and (b) PSD plot calculated by the NLDFT method of PtztpOF.

In the solid-state ^{13}C CP-MAS NMR spectrum of PtztpOF, the signals were found to appear in the expected range, indicating the formation of PtztpOF (Figure S2a). The resonance at 51 ppm indicates the bridgehead carbons of the triptycene unit in PtztpOF. Moreover, the corresponding signals for the phenyl ring and triazine ring carbons appear in the range of 120-150 ppm and 164 ppm, respectively (Figure S2a). The FTIR spectrum of the PtztpOF supports the formation of a polymer organic framework. The stretching band was found to occur at 1436 cm^{-1} for the triazine ring in PtztpOF; there was no C-Cl stretching band at 849 cm^{-1} for trichloro-triazine as a starting material.[4] The FTIR spectra of HPNC-700 and HPNC-900 provide clear evidence for the presence of surface functional groups originating from N and O hetero-atoms, as can be seen in Figure S2b. After carbonization, some fine FTIR bands of the original PtztpOF disappear in the HPNC materials. In HPNC-700 and HPNC-900, the band around 3430 $cm^{-1}$is attributed to characteristic –OH stretching vibration. The intensity of this band (~3430 cm^{-1}) in HPNC-900 is comparatively lower than that in HPNC-700 because of the higher degree of carbonization. The weak peak located at 1400 cm^{-1} is ascribed to C–N stretching vibration.[4] The XPS survey spectra demonstrate the presence of carbon, nitrogen, and oxygen species, which confirms the successful incorporation of N atoms into the as-prepared HPNCs materials by the direct one-pot carbonization of PtztpOF (Figure S2c). Although the residual nitrogen content, evaluated from XPS survey spectra, is comparatively lower in HPNC-900 (~1.94% atomic percent) than in HPNC-700 (2.71% atomic percent), the relative content of graphitic N, evaluated from the N 1s deconvoluted spectra, is more abundant in HPNC-900 (70%) than in HPNC-700 (38%) (Figure S2c and Figure S2d). These results indicate that nitrogen atoms mainly reside in the graphitic layers instead of at the periphery, which can significantly increase the electrical conductivity as well as the electrochemical response of HPNC-900 compared to those characteristics of HPNC-700. In addition, the C 1s XPS peak for HPNC-700 and HPNC-900 fitted to three sub peaks at around

284.5, 285.9, and 290.0 eV, corresponding to sp^2-hybridized C-C, C-O/C-N, and C=O, respectively (Figure S2e and Figure S2f). The above findings imply that HPNC-900 is a suitable candidate to exhibit higher electrical conductivity and electrochemical response compared to HPNC-700.

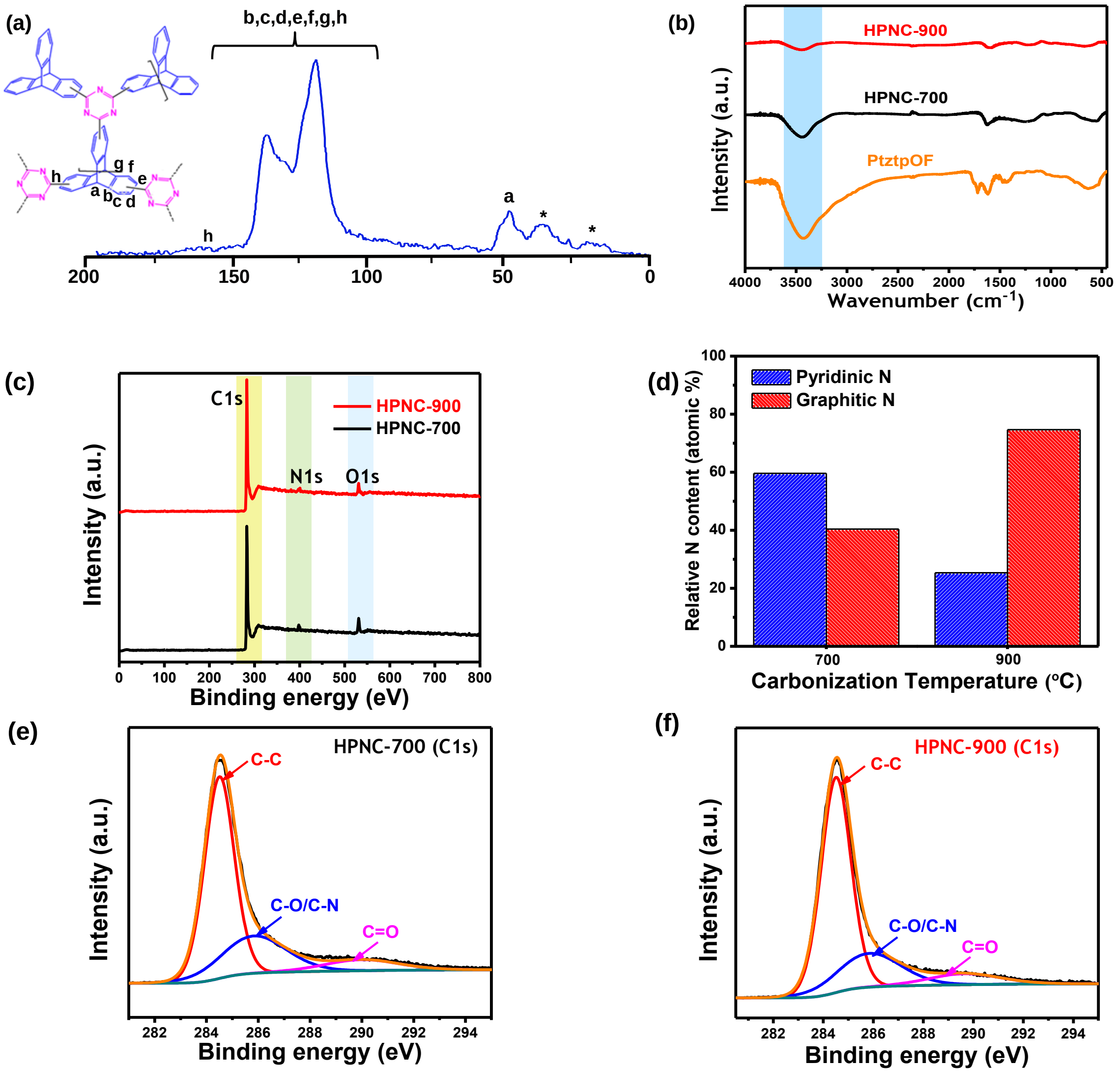

Figure S2. (a) Solid-state ^{13}C CP-MAS NMR spectrum of PtztpOF. (b) FT-IR spectra of PtztpOF, HPNC-700 and HPNC-900. (c) XPS survey spectra of HPNC-700 and HPNC-900. (d) Relative percentage contents of different types of nitrogen (graphitic and pyridinic N) for HPNCs (HPNC-700 and HPNC-900) at two different temperatures. High-resolution C 1s XPS spectra of (e) HPNC-700 and (f) HPNC-900.

Table S1. Carbon, oxygen, and nitrogen content of HPNC-700 and HPNC-900.

	Content (%)				
**Samples**	**C**	**O**	**N**	**C/O**	**C/N**
**HPNC-700**	89.78	7.51	2.71	11.95	33.13
**HPNC-900**	94.1	3.96	1.94	23.76	48.51

Figures S3a and S3b display CV curves of HPNC-700 and HPNC-900 in 1M KOH solution at varying scan rates from 10-100 mV s^{-1} in the potential window of -0.5 to +0.5 V. The rectangular shapes of the CV curves were well maintained at all scan rates, implying the ideal electrical double layer capacitor (EDLC) nature as well as the good rate capability of the HPNC materials. Interestingly, it is noted that the area under the CV curve at all scan rates is higher for HPNC-900 than it is for HPNC-700, indicating the remarkably higher specific capacitance of HPNC-900. Furthermore, the electrochemical responses for HPNC-700 and HPNC-900 in non-aqueous electrolyte (1 M EMIM-BF_4/CH_3CN) were also obtained at varying scan rates from 10-100 mV s^{-1} in the potential window of -1.0 to +1.0 V (Figure S3c,d). Although slight distortions were observed in the rectangular CV curves of both electrode materials, the shapes of the CV curves were well maintained even at high scan rate, suggesting the good rate capability of HPNC materials in non-aqueous electrolyte. These findings might be due to the strong ionic interactions between the IL, EMIM-BF_4 and the hierarchically porous nitrogen-doped carbon frameworks of the HPNCs.[5-7] Similar to the aqueous electrolyte, HPNC-900 exhibited higher specific capacitance at all scan rates compared to HPNC-700 in the 1 M EMIM-BF_4/CH_3CN electrolyte.

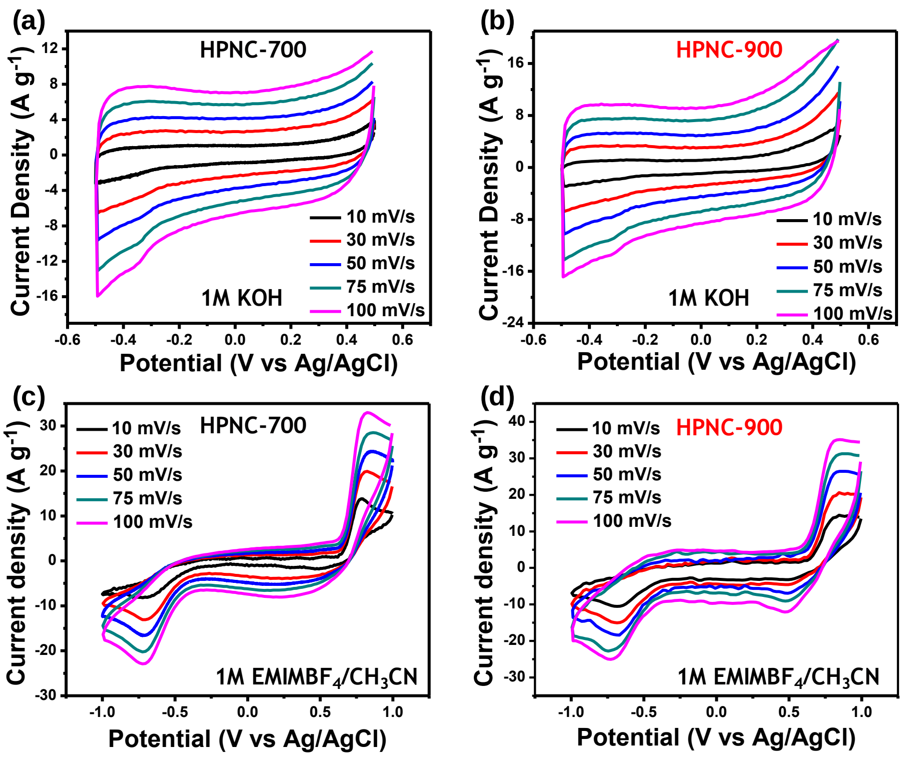

Figure S3. Electrochemical performances of HPNC based electrodes. Cyclic voltammetry analysis of (a) HPNC-700 and (b) HPNC-900 in 1 M KOH at various scan rates. Cyclic voltammetry analysis of (c) HPNC-700 and (d) HPNC-900 in 1 M EMIM-BF_4/CH_3CN electrolyte at various scan rates.

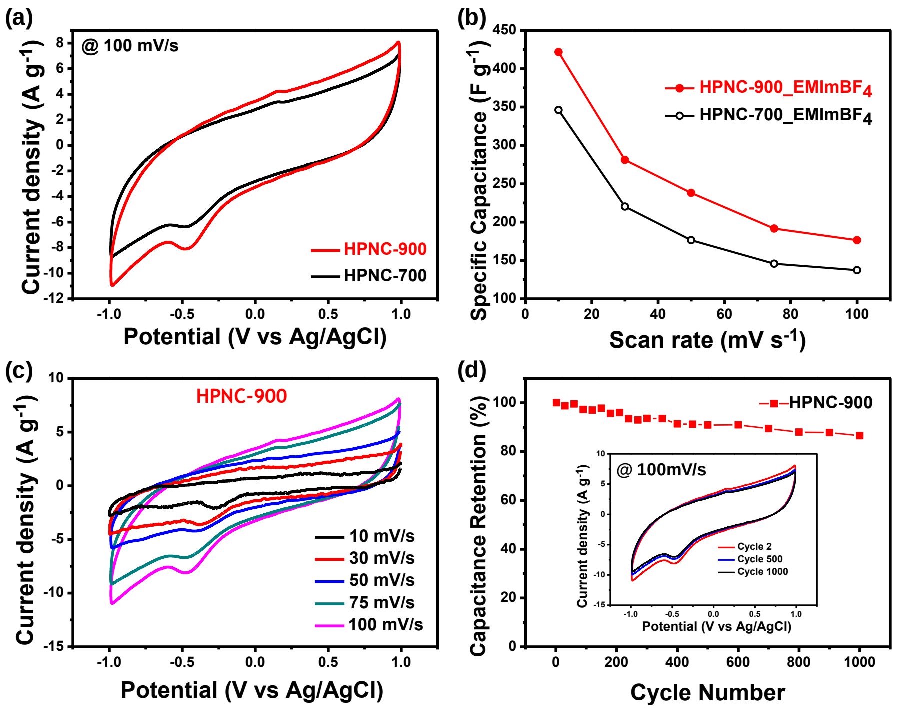

Figure S4. (a) Cyclic voltammetry analysis of HPNC-700 and HPNC-900 in EMIM-BF_4 at a scan rate of 100 mV s^{-1}. (b) Specific capacitance values of HPNC-700 and HPNC-900 at various scan rates. (c) Cyclic voltammetry analysis of HPNC-900 at various scan rate. (d) Specific capacitance retention of HPNC-900 with cycle number at a scan rate of 100 mV s^{-1}.

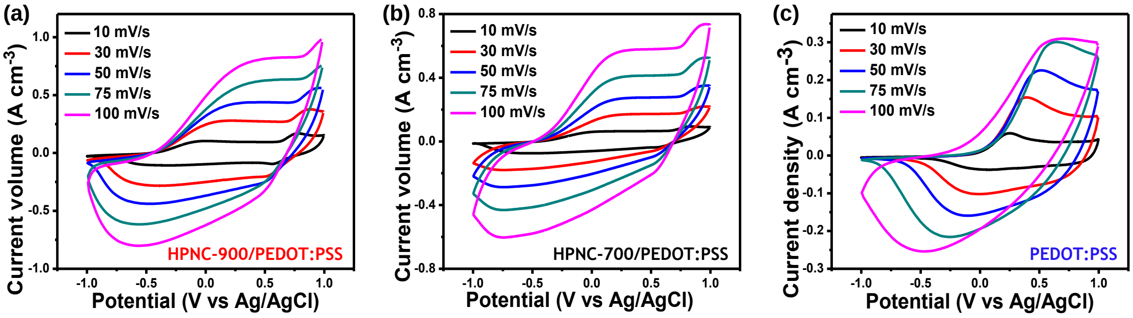

Figure S5. Electrochemical performance of HPNC based electrodes. Cyclic voltammetry analysis of (a) HPNC-900/PEDOT:PSS, (b) HPNC-700/PEDOT:PSS and (c) PEDOT:PSS electrodes in a 1 M EMIM-BF_4/CH_3CN electrolyte at various scan rates.

Table S2. Mechanical and electrical characteristics of PEDOT:PSS, HPNC-700/PEDOT:PSS, and HPNC-900/PEDOT:PSS electrodes.

Samples	Tensile Modulus (GPa)	Tensile Strength (MPa)	Elongation at break (%)	Electrical conductivity (MS m^{-1})
**PEDOT:PSS**	0.86	30.36	7.08	0.036
**HPNC-700/PEDOT:PSS**	1.21	39.35	10.02	0.058
**HPNC-900/PEDOT:PSS**	1.36	48.65	10.10	0.073

The improved electrochemical, mechanical and electrical performance of HPNCs/PEDOT:PSS is ascribed to their good compatibility of PEDOT:PSS with HPNCs through their strong interfacial interactions (Figure S6). Graphitic N introduces additional electron into the graphitic lattice of HPNCs, resulting in positively charged N atom.[8] In this context, the presence of graphitic N in HPNCs provide strong ionic interactions with adjacent sulfonate groups of PEDOT:PSS, while the presence of pyridinic N in HPNCs also leads to strong H-bonding interactions with sulfonic acid groups of PEDOT:PSS.

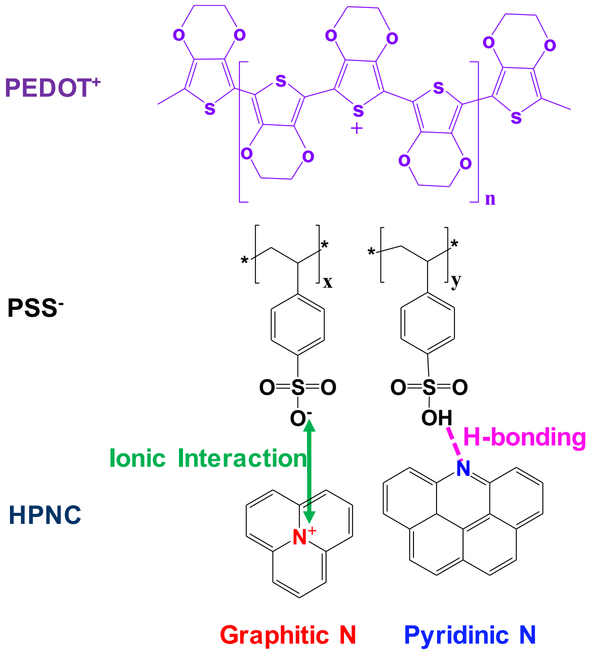

Figure S6. Interfacial interactions between PEDOT:PSS and HPNC. Ionic interactions involved between graphitic N of HPNC and adjacent sulfonate groups of PEDOT:PSS, whereas H-bonding interactions of pyridinic N of HPNC with adjacent sulfonic acid groups of PEDOT:PSS.

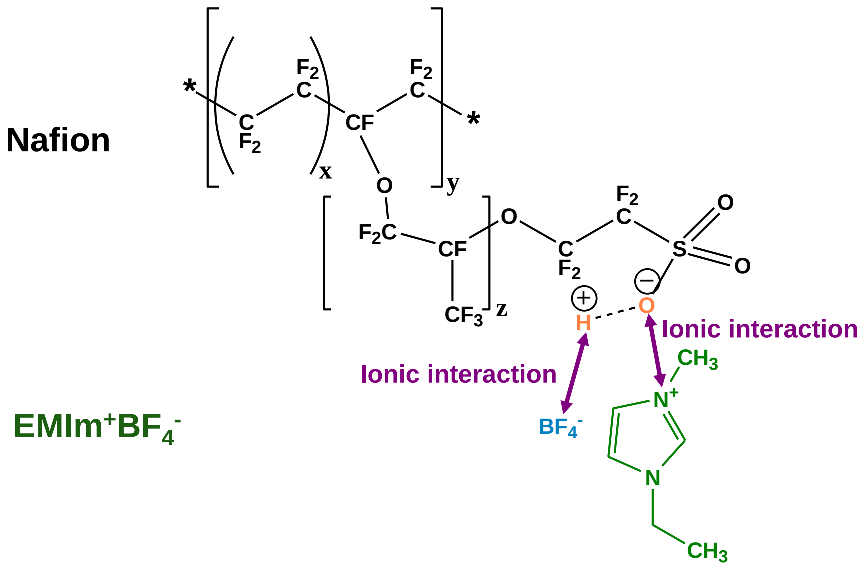

Figure S7. Chemical structures and ionic interactions of Nafion with EMIM-BF_4. The hydrophilic EMIM-BF_4 consists of $EMIM^+$ and BF_4^-, which involved strong ionic interactions with hydrophilic sulfonate (SO_3^-) and H^+ groups in Nafion.

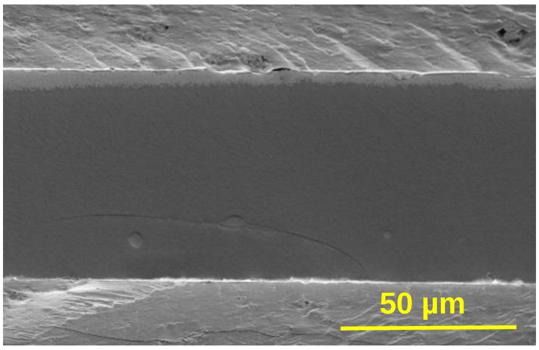

Figure S8. Cross-sectional and surface SEM image of HPNC-900/PEDOT:PSS-Nafion-IL actuator.

The energy conversion capability of an ionic type actuator, a kind of transducer, is a very important factor in evaluating actuators. Moreover, actuators with low energy consumption and high energy conversion capability are highly desirable for use in feasible applications. Therefore, to investigate the energy conversion capability of the as-fabricated actuators, current density-voltage hysteresis characteristics were studied as shown in Figure S9a. The area of the current density-voltage curve indicates the energy consumption of the actuators during a period under an input of ±0.5 V at an excitation frequency of 0.1 Hz. It is shown that the maximum current density and the *I-V* curve area of theHPNC-900/PEDOT:PSS-based actuator increases in comparison with those characteristics of the other two PEDOT:PSS and HPNC-700/PEDOT:PSS-based actuators. In addition, integrated energy consumption per electrode area values are presented in Figure S9b and Table S3; these values were obtained by calculating the area of the *I-V* curves, shown in Figure S9a. Although these results imply that theHPNC-900/PEDOT:PSS-based actuator consumed more energy than did the other actuators, the kinetic energy of HPNC-900/PEDOT:PSS-based actuator is much higher than that of the other actuators because HPNC-900/PEDOT:PSS-based actuator exhibits much larger tip displacement in comparison with the other actuators. Based on the below equation derived in ref. 9, the specific electro-mechanical energy efficiency, which refers to the energy efficiency ratio between the HPNC-900/PEODT:PSS and pure PEDOT:PSS-based actuators, is easily calculated using the energy consumption and tip displacement of the actuators, as listed in Table S3.

$$\bar{\eta}^{HPNC/PEDOT:PSS} = \frac{\eta^{HPNC/PEDOT:PSS}}{\eta^{PEDOT:PSS}} = \left(\frac{W_{Tip_{max}}^{HPNC/PEDOT:PSS}}{W_{Tip_{max}}^{PEDOT:PSS}}\right)^2 \left(\frac{\bar{E}^{PEDOT:PSS}}{\bar{E}^{HPNC/PEDOT:PSS}}\right)$$

The electro-mechanical efficiency of theHPNC-900/PEDOT:PSS-based actuator was 1.98 times and 1.79 times higher than that of the PEDOT:PSS and HPNC-700/PEDOT:PSS-based actuators, respectively. This result indicates that the conversion of electrical energy to

mechanical energy for HPNC-900/PEDOT:PSS-based actuator is better than the other actuators.

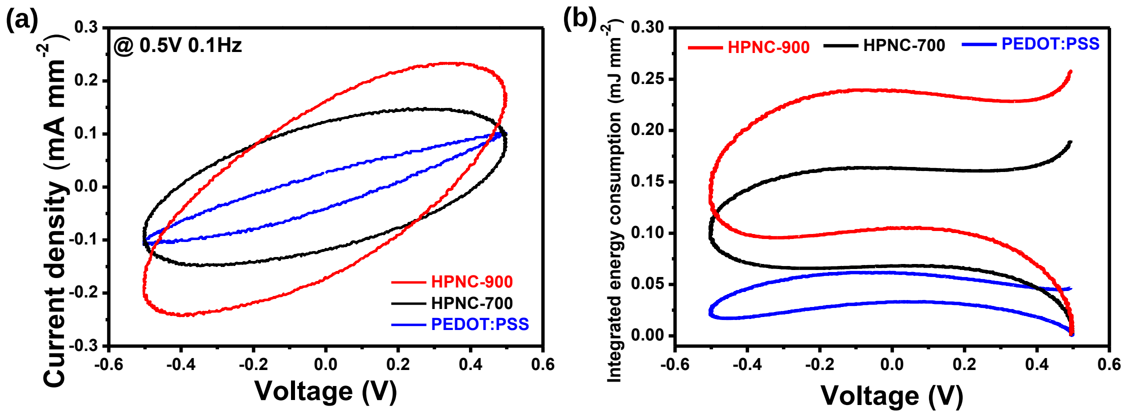

Figure S9. Current density-voltage hysteresis of the actuators under input voltage of ±0.5 V at excitation frequency of 0.1 Hz (a), and integrated energy consumption of the actuators during one-period cycle test (b).

Table S3. Specific electro-mechanical efficiency of HPNC-based actuators under input voltage of ±0.5 V at excitation frequency of 0.1Hz

**Actuators**	**Dissipated energydensity, $\overline{E}$ (mJ mm^{-2})**	**Maximum tip displacement, $W_{Tip_{max}}$ (mm)**	**Specific electro-mechanical efficiency, $\overline{\eta}^{\text{nano-composite}}$**
**PEDOT:PSS actuator**	0.047	1.062	1
**HPNC-700/PEDOT:PSS actuator**	0.190	2.241	1.101
**HPNC-900/PEDOT:PSS actuator**	0.258	3.497	1.975

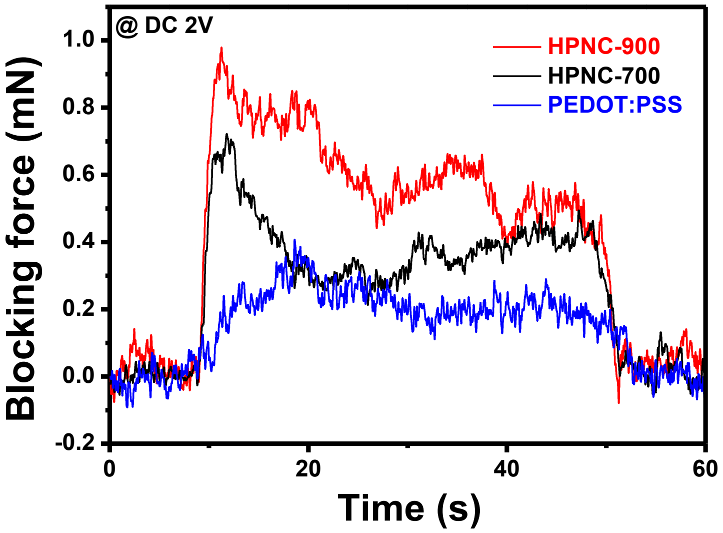

Figure S10. Blocking forces of all actuators under the DC input of 2V.

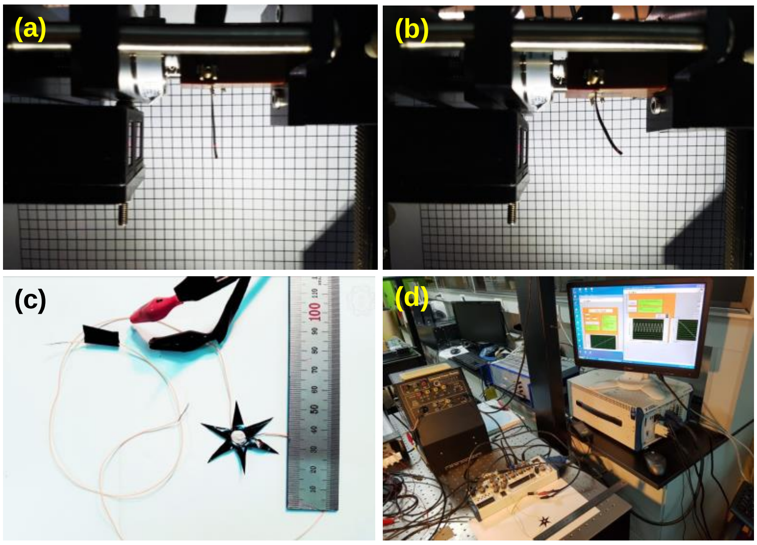

Figure S11. The photographs of the experimental setup for actuation tests: (a) Before actuation, (b) Bending actuation under electrical stimulus, (c) Dimension of a flower-like actuating device and (d) the setup for actuation tests including a NI-PXI data acquisition system and a current amplifier.

References

[1] P. Pandey, A. P. Katsoulidis, I. Eryazici, Y. Wu, M. G. Kanatzidis, S. T. Nguyen, *Chem. Mater.* **2010**, *22*, 4974.

[2] A. P. Katsoulidis, S. M. Dyar, R. Carmieli, C. D. Malliakas, M. R. Wasielewski, M. G. Kanatzidis, *J. Mater. Chem. A* **2013**, *1*, 10465.

[3] J. Wang, I. Senkovska, M. Oschatz, M. R. Lohe, L. Borchardt, A. Heerwig, Q. Liu, S. Kaskel, *ACS Appl. Mater. Interfaces* **2013**, *5*, 3160.

[4] S. Dey, A. Bhunia, D. Esquivel, C. Janiak, *J. Mater. Chem. A* **2016**, *4*, 6259.

[5] N. Guo, M. Li, Y. Wang, X. Sun, F. Wang, R. Yang, *ACS Appl. Mater. Interfaces* **2016**, *8*, 33626.

[6] M. Kotal, J. Kim, K. J. Kim, I. K. Oh, *Adv. Mater*. **2016**, *28*, 1610.

[7] Z. Wen, X. Wang, S. Mao, Z. Bo, H. Kim, S. Cui, G. Lu, X. Feng, J. Chen, *Adv. Mater.* **2012,** *24*, 5610.

[8] Y. C. Lin, P. Y. Teng, C. H. Yeh, M. Koshino, P. W. Chiu, K. Suenaga, *Nano Lett.* **2015**, *15*, 7408.

[9] J. H. Jung, J. H. Jeon, S. Vadahanambi,I. K. Oh, *Carbon* **2011**, *49*, 1279.
